# The Role of Infection in the Pathogenesis of Vaso-Occlusive Crisis in Patients with Sickle Cell Disease.

**DOI:** 10.4084/MJHID.2011.028

**Published:** 2011-07-08

**Authors:** Sagir G. Ahmed

**Affiliations:** Department of Haematology, Aminu Kano Teaching Hospital

## Abstract

Sickle cell disease (SCD) is characterized by recurrent vaso-occlusive crisis (VOC). Patients with SCD have impaired immunity and are thus predispose to infections. The vast majority of SCD patients live in underdeveloped nations with high prevalence and transmission rates of infections. This makes the SCD patients prone to infections, which frequently precipitate VOC. We reviewed the role of infection in the pathogenesis of VOC, taking into consideration all potential mechanisms from previous studies and hypothetical perspectives. The potential mechanisms through which infections may lead to VOC involve several pathological changes including pneumonitis, pyrexia, acute phase reaction, hypercoagulability, neutrophilia, eosinophilia, thrombocytosis, bronchospasm, red cell cytopathic and membrane changes, auto-antibodies mediated red cell agglutination and opsonization, diarrhoea and vomiting, which may act singly or in concert to cause red cell sickling. These changes can induce sickling directly or indirectly through their adverse effects on Hb oxygenation and polymerization, hydration, blood viscosity, red cell metabolism, procoagulant activation, intercellular adherence and aggregation, culminating in VOC. There is therefore the need to ameliorate the burden of infection on SCD through immunization, prophylactic and therapeutic use of antimicrobials, barrier protection and vector control in communities with high prevalence of SCD.

## Introduction:

Haemoglobin S (HbS) is a structural variant of the normal haemoglobin (HbA) and is due to a genetic mutation in the β globin gene where thymidine replaced adenine resulting in the substitution of glutamic acid by valine in position 6 of the β-globin chain.^1^ This substitution caused a significant alteration in the physico-chemical properties of HbS, which has a reduced solubility in the deoxygenated state.^2^ The sickle β-gene mutation confers relative protection against falciparum malaria among individuals with sickle cell trait (SCT).^3^,^4^ Consequently, through the process of natural selection, children with SCT have relative higher survival advantage in malaria endemic zones.^3^,^4^ This phenomenon is responsible for the high prevalence of SCT in the malaria endemic zones of Africa where up to 10–40% of indigenous populations have SCT.^5^ Therefore, malaria is the single most important infective driver for the perpetuation of SCT, which, through carrier inter-marriages, had led to a high prevalence of sickle cell disease in black Africa.^1^,^3^,^5^ While homozygous HbS disease (sickle cell anemia) is the most common type of sickle cell disease (SCD), less common types of SCD arise as a result of double heterozygosity between HbS gene and different β-globin gene mutations such as haemoglobin C (HbSC) or β-thalassaemia (HbSβthal) that share a similar basic pathophysiology.^1^,^2^,^6^ The clinical presentation of SCD is due to vaso-occlusive episodes resulting from polymerization of deoxygenated Hb-S leading to the formation of sickled red cells.^2^ The clinical course of SCD is typically characterized by variable periods of steady state that is periodically interrupted by painful vaso-occlusive crisis, which can be triggered by psychological, physical and infective factors.^7^,^8^

Patients with SCD have increased susceptibility to infections, which is partly due to autosplenectomy resulting from recurrent vaso-occlusive infarcts within the spleen.^9^ Several other factors that predispose the SCD patients to infections have also been reported, which include abnormalities of opsonization, antibody production, the alternate complement pathway, leukocyte functions and cell-mediated immunity.^10^,^11^,^12^ Consequently, life-threatening infections are major causes of morbidity and mortality in patients with SCD. The range of immune abnormalities in SCD to a large extent determines the pattern of microbiological susceptibility in affected patients. Thus hyposplenism predisposes to severe infections with malaria and encapsulated organisms including *Haemophilus influenza and Streptococcus pneumoniae*, while low serum IgM levels, impaired opsonization, and abnormality of complement pathway would further increase susceptibility to other common infectious agents, including *Mycoplasma pneumoniae*, *Salmonella typhimurium*, *Staphylococcus aureus,* and *Escherichia coli*.^9^,^13^ In addition to immunological dysfunction, another factor that increases susceptibility to bacterial infection in patients with SCD is recurrent tissue infarcts. Tissue infarcts provide potential primary foci for infections that are easily propagated within the context of a pre-existing immunological dysfunction associated with the background SCD.^14^ Chronic haemolysis and vasculopathy are major manifestations of SCD.^15^ Hence, another factor that increases the susceptibility of SCD patients to infection is chronic transfusion therapy, which has gained prominence in the prevention and management of stroke, priapism, pulmonary hypertension, acute chest syndrome and chronic renal failure in affected patients in whom iron overload has become increasingly common.^16^ However, it should be appreciated that iron overload in SCD patients will raise the risk of infections with iron-dependent bacteria such Yersinia species, thereby amplifying the pre-existing risk of infection due to the background immunodeficiency associated with SCD.^17^ Precious studies had demonstrated that malaria, bacterial and other forms of infections are associated with crises, exacerbation of morbidity and poor survival among patients with SCD.^8^,^18^

Red cell sickling is a pathognomonic feature of SCD. Red cells of SCD patients go through repeated cycles of deoxygenation (in the tissues) and re-oxygenation (in the lungs). This sequence of events creates a dynamic scenario of sickling and un-sickling until the cell membrane sustains a significant degree of damage, which eventually leads to the formation of irreversibly sickled cells that are invariably haemolysed.^19^ Hence SCD is associated with chronic haemolysis, which is consistently accompanied by reticulocytosis. Sickle reticulocytes had been shown to abundantly express the alpha-4 beta-1 integrin complex, which binds endothelial VCAM-1 receptors.^20^ Therefore, sickle reticulocytes endothelial adhesion is thought to play a primary role in the initiation of vascular occlusion in the pathophysiology of VOC, which is subsequently amplified by continued red cell sickling and piling of irreversibly sickled cells due to hypoxia and/or other sickling-inducing factors.^21^ However, red cell sickling is more prominent during crisis, but continuous sickling does occur at a lower rate even in the steady state.^22^ Therefore, any causative factor for VOC must necessarily be able to significantly increase the rate of sickling and/or decrease the rate of un-sickling (i.e. reversal of reversibly sickled cells to discocytes) to a level that would significantly shift the clinical status of the patient from steady state to crisis.

In this report we reviewed the potential mechanisms by which infection can contribute to the pathogenesis of VOC in patients with SCD. These mechanisms involve pathological changes including pneumonitis, pyrexia, acute phase reaction, hypercoagulability, neutrophilia, eosinophilia, thrombocytosis, bronchospasm, red cell cytopathic and membrane changes, auto-antibodies mediated red cell agglutination and opsonization, diarrhoea and vomiting, that may act singly or in concert to cause increased sickling and/or decrease un-sickling and potentially culminate in VOC as outlined in Table 1.

## Pneumonic and Inflammatory Changes with Impaired Gaseous Exchange:

Respiratory tract infections in patients with SCD vary in severity from mild upper tract infection to moderately severe uncomplicated pneumonia that can be managed with appropriate antibiotics. Nonetheless, every case of respiratory tract infection must be monitored closely because of the potential risk of acute chest syndrome (ACS), which is a serious and potentially fatal complication.^23^ The commonest cause of the ACS is acute pulmonary infection by a community-acquired pathogens, which incite pneumonic changes due to excessive inflammatory response to what often should have been a mild respiratory infection in patients without SCD.^23^ The susceptibility of patients with SCD to develop excessive pulmonary inflammatory response and infiltrates was corroborated by previous studies, which had shown that transgenic mice with HbS were highly susceptible to inflammatory triggers such as endotoxins and environmental hypoxia with the development of pulmonary tissue injury at doses that would not adversely affect wild-type non-transgenic mice.^24^,^25^ Therefore, it can be deduced that SCD confers upon its sufferers special propensity for exaggerated pulmonary inflammatory response that can transform apparently simple acute chest infections to ACS. ACS is associated with intense alveolar consolidation and pulmonary sequestration of sickled red cells, resulting in lung injury and impaired gaseous exchange across the alveolar membrane.^23^ These pulmonary changes would predictably lead to a fall in PaO_2_, generalized hypoxia, increased red cell sickling, decreased pulmonary conversion of deoxy-HbS to oxy-HbS, failure of reversal of sickled red cells to discocytes, piling of sickled cells and VOC. Hence, ACS can be rapidly progressive and fatal unless managed with blood gas monitors, oxygen therapy and exchange blood transfusion in addition to appropriate antibiotics administration.^26^

It should be appreciated that chronic chest infections can also interfere with pulmonary function and intensify red cell sickling in SCD patients. This is particularly important because the vast majority of SCD patients live in underdeveloped nations with high prevalence and transmission rates of tuberculosis within the general populations.^27^ Patients with SCD are at high risk of contracting tuberculosis infection in view of their impaired immunity.^10^,^11^,^12^,^27^ Previous study had shown that pulmonary tuberculosis in SCD patients was associated with increased red cell sickling, which was a reflection of impaired pulmonary function and hypoxia.^28^ Hence, pulmonary dysfunction due to chronic chest infections would increase the risk of VOC in patients with SCD.^28^

It is therefore imperative that SCD patients with acute or chronic infections of the lungs must be closely monitored with blood gas analyzers so as to detect and correct hypoxia and its deleterious effect on red cell sickling.

## Acute Phase Reaction:

Vascular occlusion and tissue necrosis, though more pronounced during VOC, also occur at lower rate during the steady state.^22^ Earlier studies had revealed that re-perfusion of necrotic tissues resulted in the generation of oxygen radicals, leading to inflammatory endothelial and tissue injury.^29^,^30^ Many inflammatory markers of acute phase reaction are elevated in SCD patients even in steady state, including C-reactive protein, TNF-alpha, and interleukin-1 and -8, in addition to the mediators of endothelial activation such as VCAM-1 and endothelin-1.^31^ Continuous inflammation and generation of oxygen radicals would lead to a high utilization of antioxidant reserves in patients with SCD. Consequently, some studies had shown that patients with SCD in steady state had significantly reduced total antioxidant status.^32^ Further more, it had been revealed that the risk of developing VOC correlated negatively with the levels of total antioxidant status, suggesting that inflammation associated oxidative stress contributes to the pathophysiology of VOC.^32^ These reports clearly under scored the fact that SCD is by itself a state of systemic chronic inflammatory disorder even in the absence of infection. However, the acquisition of infection by patients with SCD would certainly aggravate the background inflammatory response and generate more oxygen radicals through oxidative respiratory bursts of activated neutrophils and phagocytes, which will cause further depletion of antioxidant reserves and precipitate VOC.^29^,^30^,^32^,^33^ It is therefore reasonable to continue to investigate the potential usefulness of antioxidants in mitigating red cell sickling, prevention and attenuation of VOC in SCD patients with infection.^34^ Further more, it should be appreciated that iron overload, in addition to predisposing to infection, is associated with increased generation of oxygen free radicals that would deplete antioxidant stores and aggravate inflammatory cellular injuries in patients with SCD.^35^ Therefore, chronic transfusion therapy in SCD must be used judiciously with the complement of iron chelation therapy in order to mitigate the deleterious effect of iron overload.^36^

Continuing infection would trigger humoral response with immunoglobulin production while ensuring a sustained elevation of plasma levels of acute phase reactants including fibrinogen, FVIII, vWF and other coagulation factors that would raise plasma viscosity and induce hypercoagulability.^37^ Sustained hyperviscosity would result in stasis and increased production of deoxy-HbS with tendency towards red cell sickling, while hypercoagulability would lead to intra-vascular fibrin deposition thereby reinforcing the vascular occlusion.^38^ Yet other inflammatory makers such as VCAM-1 and ICAM-1 induce adhesion of sickled red cells onto the endothelium, which further jeopardizes vascular patency.^39^

It is therefore obvious that changes in plasma proteins due to infection and inflammatory acute phase reaction can adversely affect rheology, coagulability, intercellular adhesions and antioxidant reserves, all of which significantly raise the risk of red cell sickling and VOC in patients with SCD. Hence, it is of paramount importance that infections in SCD must be treated at the outset without delay in order to preempt the intensity of acute phase reaction and their vaso-occlusive effects.

## Pyrexia, Diarrhoea, Vomiting:

Pyrexia could arise as a result of the pyrogenic effects of some inflammatory acute phase reactants such as TNF-alpha (endogenous pyrogens) or as a result of the effect of exogenous pyrogens associated the infecting micro-organisms.^40^ In addition to pyrexia, enteropathogenic organisms such as E Coli, Salmonella and Shigella species could cause severe diarrhea and vomiting.^41^ Pyrexia is invariably associated with increased rate of perspiration and water loss via the skin, while diarrhea and vomiting would result in gastro-intestinal water loss. The combined effect of pyrexia, diarrhea and vomiting would lead to dehydration, hyperviscosity and stasis. Patients with SCD are particularly susceptible to dehydration due to their inability to conserve water as a result of hyposthenuria.^42^ Hence, through the concerted effect of acute phase reaction and dehydration, leading to hyperviscosity and stasis, infection can be an efficient trigger of VOC in patients with SCD. In addition to its role in causing dehydration, pyrexia is particularly deleterious in patients with SCD as previous studies had shown that rates of HbS polymerization and red cell sickling were faster at higher temperatures.^43^,^44^ Further more, sustained uncorrected plasma dehydration would lead to red cell dehydration and elevation of MCHC, which is an important promoter of HbS polymerization and red cell sickling.^43^,^44^

It can therefore be surmised that the triple concert between pyrexia, diarrhoea and vomiting in a patient with SCD would result in dehydration and impaired rheology, which will cause stasis and increased production of deoxy-HbS, the polymerization of which is subsequently enhanced by high temperature and rising MCHC eventually leading to sickling and VOC. This scenario under scores the indispensible roles of adequate intravenous hydration and antipyretics, in addition to antibiotics, in the management of SCD patients with infections.

## Neutrophilia:

Neutrophilia is a useful marker of infection in many clinical settings. However, it must be appreciated that modest neutrophilia is a common feature of SCD even in steady state in the absence of infection. Steady state neutrophilia was thought to be due to redistribution of neutrophils from marginal to circulating pool.^45^ Nonetheless, the prevalence and intensity of neutrophilia are higher in SCD patients with bacterial infections in comparison to those without infection.^28^,^46^ In our experience, the neutrophilia in SCD patients with bacterial infections was usually accompanied by eosinopenia, which was usually absent in non-infected patients.^28^,^46^ Eosinopenia of infection is mediated by inflammatory response associated with the release of adrenal corticosteroids, epinephrine and chemotactic factors, which lead to rapid peripheral sequestration of eosinophils and their migration into inflammatory sites.^47^,^48^ Neutrophilia in SCD patients with infection would have far reaching consequences with respect to the pathophysiology of sickling. Firstly, as earlier mentioned, infectious activation of neutrophils would trigger respiratory bursts leading to generation of oxygen free radicals, depletion of antioxidant reserves and predispose to sickling and VOC.^32^,^33^ Secondly, increased number of circulating activated neutrophils would lead to greater oxygen consumption, which may cause hypoxia, resulting in sickling and VOC.^33^,^49^ Thirdly, neutrophilia would aggravate blood viscosity, which will lead to stasis and increased production of deoxy-HbS and red cell sickling.^50^ And fourthly, neutrophils in SCD exhibit increased adherence to endothelium and sickle red cells, hence, neutrophilia would promote vascular occlusion in patients with SCD.^51^,^52^

It is therefore not surprising that the beneficial role of hydroxyurea in the management of SCD is partly related to its ability to cause modest reduction in the number of circulating neutrophils, thus counterbalancing their adverse role in the pathogenesis of vascular occlusion.^53^

## Thrombocytosis:

Thrombocytosis is a common finding in patients with SCD even in steady state in the absence of any infection. This phenomenon was attributed to the background haemolytic anaemia and autosplenectomy.^22^,^27^,^54^ However, previous studies revealed that SCD patients with infections had higher platelet count and more intense thrombocytosis in comparison to their counterparts without infections.^28^ This finding was interpreted to be a reflection of additional effect of reactive inflammatory changes associated with infection.^28^,^55^ In similarity to neutrophilia, thrombocytosis can raise blood viscosity and predispose to stasis, sickling and VOC.^50^ The hallmark of VOC is tissue necrosis, which is associated with vascular endothelial damage and dysfunction.^56^,^57^,^58^ Endothelial damage leads to exposure of subendothelial structures, including sub-endothelial microfibrils and collagen, both of which cause platelet activation and aggregation resulting in vascular occlusion.^56^,^57^,^58^

It can therefore be surmised that any infections in patients with SCD would lead to intensification of thrombocytosis and increase the risk of VOC. It is within the context of these findings that some researchers are exploring the possible role of anti-platelet agents in mitigating vaso-occlusive complications of SCD.^59^

## Erythrocytopathic Effect of Malaria:

The SCT offers relative protection against malaria infection.^60^ The mechanism of protection was thought to be largely related to innate factors such as the reduced ability of Plasmodium falciparum parasites to grow and multiply in SCT red cells.^60^ Recent studies have suggested that protection against malaria in SCT might also involve the accelerated acquisition of malaria-specific immunity within the context of the normal immune response in persons with SCT.^61^,^62^ In contradistinction, patients with SCD have impaired immune response and are prone to develop infections including severe malaria, which was reported to be the most common trigger of VOC in patients living in malaria endemic countries.^18^,^63^ Malaria parasites directly invade and replicate within red cells during the erythrocytic phase of its lifecycle. Infected red cells would invariably sickle as a result of metabolic changes induced by the invading parasites.^64^ The sickled red cells subsequently adhere to vascular endothelium via ICAM-1 and VCAM-1 receptors.^39^ The potency of malaria infection in inducing VOC may be related to its special ability to deform the membrane of infected red cells leading to the formation of histidine rich protein knobs.^65^ These knobs confer upon malaria infected sickled red cells the affinity for vascular endothelium leading to adhesion.^65^ Therefore, the malaria infected sickled red cell has double predilection for the endothelium due to expression of cytoadhesion molecules (VCAM-1 and ICAM-1) on the endothelium and due to the cytoadherent effect of the histidine rich protein domains in the knobs on the red cell membrane.^39^,^65^ This double predilection makes the malaria infected sickled cell extremely adherent to the endothelium. We therefore deduce that the dual ability of malaria to directly induce sickling of red cells (by red cell invasion) and potentiate their adherence to endothelium (by Knob formation) are the principal factors that make malaria infection the most common and potent trigger of VOC in SCD patients in malaria endemic countries. This underscores the importance of continuous lifelong anti-malarial prophylaxis in the management of SCD patients living in malaria endemic zones.^63^

## Loffler’s and Tropical Pulmonary Eosinophilia Syndromes:

The vast majority of patients with SCD live in tropical and under-developed nations of the world with high prevalence of protozoan and helminthic infections that are related to poor personal and environmental sanitation and hygiene. It is therefore important to address the possible role of these infections with respect to VOC. Nonetheless, there is paucity of literature with regards the role of intestinal parasites as co-morbid factors in patients with SCD in steady state and in the induction of VOC. An isolated study from the middle east had demonstrated that patients with SCD in steady state had relatively higher prevalence of protozoan and helminthic intestinal parasites, which was attributed to their immune-compromised status.^66^ A solitary uncontrolled case-study from Nigeria had reported high prevalence of intestinal helminthic infections among SCD patients in VOC, suggesting a causal relationship.^67^ But the study did not expound on the pathologic mechanisms through which the intestinal parasites could have triggered the VOC.

We infer that intestinal parasites may trigger VOC by direct affectation of the lungs and impairment of oxygenation during the pulmonary migratory phase as seen in Loffler’s syndrome commonly associated with ascariasis and ancylostomiasis.^68^,^69^ Loffler’s syndrome typically presents with parasitic and eosinophilic pulmonary infiltrates, consolidations and reactive bronchospasm.^68^,^69^ These pathological processes that are associated with Loffler’s syndrome can eventually lead to a fall in PaO_2_, generalized hypoxia, increased red cell sickling, decreased pulmonary conversion of deoxy-HbS to oxy-HbS, failure of reversal of sickled red cells to discocytes, piling of sickled cells and VOC. In addition, the peripheral blood eosinophilia, which is a regular accompaniment of Loffler’s syndrome is also a risk factor for the development of VOC since activated eosinophils have been shown to adherence to vascular endothelium and contribute in the pathogenesis of VOC.^70^

Filariasis is another common tropical disease that causes Tropical Pulmonary Eosinophilia, which is characterized by microfilarial pulmonary infiltrates, bronchospasm and peripheral blood eosinophilia.^71^ In similarity to Loffler’s syndrome, tropical pulmonary eosinophilia can cause generalized hypoxia, increased red cell sickling with decreased pulmonary conversion of deoxy-HbS to oxy-HbS, failure of reversal of sickled red cells and increased eosinophil adherence to vascular endothelium, all of which can precipitate VOC. Hence, in parasite endemic zones, Loffler’s and tropical pulmonary eosinophilia syndromes must be entertained as differential diagnoses in SCD patients presenting with clinical features of pneumonia, ACS or asthmatic episodes with or without VOC; and relevant stool, skin and blood tests must be conducted to confirm or rule them out. It is therefore rational to infer that any parasitic infection that affects the lung or causes sustained eosinophilic change in the lungs and/or the peripheral blood is a risk factor for VOC in patients with SCD.

It is imperative that patients with SCD in parasites endemic zones should be regularly subjected to screening tests for early detection and treatment of parasitic diseases during routine clinic visits.

## Mycoplasma Infection and Red Cell Auto-antibody:

One of the most important atypical causative agent of ACS in patients with SCD is *Mycoplasma pneumoniae*, which is particularly common in young children.^72^ Mycoplasma infection can be complicated by the development of complement fixing IgM anti-I cold reacting red cell auto-antibodies that can cause agglutination and haemolysis in the colder peripheral parts of the body.^73^ More over, in rare cases mycoplasma infection maybe associated with warm IgG red cell auto-antibodies that can cause agglutination and haemolysis even at core body temperature of 37°C.^74^ Therefore, SCD patients who develop ACS due to mycoplasma are at risk of developing increased haemolysis if the infection is associated with red cell auto-antibodies.^73^ We infer that auto-agglutination in patients with SCD will also produce large red cell aggregates that would predictably result in increased blood viscosity, stasis and impairment of blood flow leading to increased production of deoxy-HbS, red cell sickling and VOC. Auto-antibodies can also cause opsonization of sickled red cell, which would predictably lead to increased adherence of opsonized sickle cells to Fc and C3 complement receptors that exist on membranes of neutrophils.^75^ This adherence effect of opsonins will aggravate the well established role of neutrophil-sickle cell interactions in VOC as reported previously.^51^,^52^ Hence, we hypothetically infer that red cell auto-antibodies would increase the risk of VOC in SCD through the processes of agglutination (that increases viscosity) and opsonization (that increases sickled cell-neutrophil adhesion).

There is therefore the need for detailed studies to evaluate the incidence of auto-antibodies and their effect on rheology, sickling, opsonization, neutrophil-sickle cell adherence and vascular occlusion in SCD patients infected by mycoplasma. Meanwhile, it is important to ensure that SCD patients with ACS and mycoplasma infection are promptly screened for cold and warm auto-agglutinins. While keeping the patients in warm environment will largely mitigate the effect of cold antibodies, cautious administration of steroidal immunosuppressive agents maybe necessary to abolish the production of warm auto-antibodies while taking care not to further jeopardize the inherent immune dysfunction due to the background SCD.

## Conclusion and Recommendation:

Infectious diseases have a multitude of proven and hypothetical mechanisms through which they can cause red cell sickling and precipitate VOC in patients with SCD. The role of infections in the pathogenesis of VOC is a particularly serious and challenging one in view of the background immunodeficiency associated with SCD and the high prevalence of infectious diseases in the underdeveloped countries within which most patients with SCD live. It is therefore imperative for governmental authorities and clinicians in countries with SCD to work concertedly to reduce the burden of infection on SCD patients. This can only be achieved through sustainable immunization programs, effective prophylactic and therapeutic use of antimicrobial agents, widespread use of barrier protections such as insecticide-treated mosquito nets and the introduction of environmental vector control measures for parasitic diseases. Poverty and illiteracy are also prevalent in the underdeveloped nations of the world. Hence, eradication of poverty and illiteracy, as enshrined in the United Nations’ millennium development goals for underdeveloped countries, is a necessary requirement for curtailing the high prevalence of infection and improving the quality of life of SCD sufferers in the long term.

## Figures and Tables

**Table 1:** Possible Mechanisms for VOC in SCD Patients with Infections

**Pathological Processes**	**Common Causative Pathogens**	**Possible Mechanisms Leading to Red Cell Sickling & VOC**
Chest Infection/Pneumonic consolidations/Acute chest syndrome.	Acute bacterial. Mycoplasma. Tuberculosis.	Inflammatory infiltrates leading to impaired oxygenation. Peripheral blood hypoxia generates more sickled cells. Decreased conversion of deoxy-HbS to oxy-HbS. Accumulation of deoxy-HbS. Failure to convert reversibly sickled cells to discocytes.
Acute phase reaction.	Any infection.	Increased plasma viscosity. Decreased capillary flow/stasis. Increased production of deoxy-HbS.
Pyrexia, Diarrhoea, Vomiting.	Any infection. Especially enteropathogens.	Excess water loss leads to dehydration. Dehydration leads to high plasma viscosity and osmolality, stasis, increased production of deoxy-HbS. Dehydration leads to high MCHC. High MCHC increases rate of red cell sickling High temperature increases rate of HbS polymerization and sickling.
Neutrophilia.	Pyogenic bacteria. Tuberculosis.	Increased blood viscosity/stasis lead to increased production of deoxy-HbS. Increased oxygen consumption by activated neutrophils lead to increased production of deoxy-HbS. Activated neutrophils generate more free radicals, decrease antioxidants and predispose to red cell sickling. Increased adhesion of neutrophils to sickled cells and vascular endothelium lead to vascular occlusion.
Thrombocytosis.	Most infections, except malaria and some viruses.	Increased blood viscosity/stasis lead to increased production of deoxy-HbS. Platelets activated by endothelial damage leading to vascular occlusion.
Erythrocytopathic effect of malaria.	Plasmodium falciparum.	Malaria invasion of red cell induces massive sickling and KAHRP knobs formation on red cell membrane. Sickling induce formation of ICAM-1 and VCAM-1 on vascular endothelial membrane. Formation of KAHRP knobs on red cells enhance adhesion of infected sickled red cells to endothelium and promotes vascular occlusion. KAHRP knobs act synergistically with endothelial ICAM-1 and VCAM-1 receptors to enhance sickle cell adherence to endothelium.
Loffler’s syndrome.	Ascariasis and Ancylostomiasis.	Migrating larvae/eosinophilic infiltrates in the lungs and bronchospasm leading to impaired oxygenation. Peripheral blood hypoxia generates more sickled cells. Decreased conversion of deoxy-HbS to oxy-HbS. Accumulation of deoxy-HbS. Failure to convert reversibly sickled cells to discocytes. Peripheral eosinophilia with activated eosinophils adhering to endothelium and causing vascular occlusion.
Tropical pulmonary eosinophilia syndrome.	Filariasis.	Microfilarial/eosinophilic infiltrates in the lungs and bronchospasm leading to impaired oxygenation. Peripheral blood hypoxia generates more sickled cells. Decreased conversion of deoxy-HbS to oxy-HbS. Accumulation of deoxy-HbS. Failure to convert reversibly sickled cells to discocytes. Peripheral eosinophilia with activated eosinophils adhering to endothelium and causing vascular occlusion.
Red cell autoantibody formation.	Mycoplasma pneumoniae.	Auto-antibodies cause red cell agglutinations & stasis, increased production of deoxy-HbS and sickling. Auto-antibodies cause sickled red cell opsonization. Increased adhesion between opsonized sickled red cell and neutrophil via Fc & C3 receptors, leading to vascular occlusion.

KAHRP=Knob Associated Histidine Rich Proteins.
